# Enabling relationship formation, development, and closure in a one-year female mentoring program at a non-governmental organization: a mixed-method study

**DOI:** 10.1186/s12889-016-2850-2

**Published:** 2016-02-24

**Authors:** Madelene Larsson, Camilla Pettersson, Therése Skoog, Charli Eriksson

**Affiliations:** Faculty of Medicine and Health, School of Health Sciences, Örebro University, Örebro, 701 82 Sweden; School of Law, Psychology and Social Work, Örebro University, Örebro, 701 82 Sweden

**Keywords:** Mixed methods, Mentoring, Gender, Intervention, Emerging adulthood, Young women, Prevention, NGO, Relationship process, Sweden

## Abstract

**Background:**

Mental health problems among young women aged 16–24 have increased significantly in recent decades, and interventions are called for. Mentoring is a well-established preventative/promotive intervention for developing adolescents, but we have yet to fully understand how the relationship between the mentor and the protégé forms, develops, and closes. In this study, we focused on a female mentoring program implemented by a Swedish non-governmental organization, The Girls Zone. First, we examined the psychological and social characteristics of the young women who chose to take part in the program as protégés. Second, we investigated adolescent female protégés’ own experiences of the relationship process based on a relational-cultural theory perspective.

**Methods:**

The mixed-method study included 52 questionnaires and five semi-structured interviews with young women aged 15–26 who had contacted The Girls Zone between 2010 and 2012 in order to find a mentor. Their experience of the mentoring relationships varied in duration. Data were analysed statistically and with inductive qualitative content analysis.

**Results:**

The group of protégés was heterogeneous in that some had poor mental health and some had good mental health. On the other hand, the group was homogenous in that all its members had shown pro-active self-care by actively seeking out the program due to experiences of loneliness and a need to meet and talk with a person who could listen to them. The relationships were initially characterized by feelings of nervousness and ambivalence. However, after some time, these developed into authentic, undemanding, non-hierarchical relationships on the protégés’ terms. The closure of relationships aroused feelings of both abandonment and developing strength.

**Conclusions:**

Mentorships that are in line with perspectives of the relational-cultural theory meet the relationship needs expressed by the female protégés. Mentor training should focus on promoting skills such as active listening and respect for the protégé based on an engaged, empathic, and authentic approach in a non-hierarchical relationship. These insights have the potential to inform interventions in several arenas where young women create authentic relationships with older persons, such as in school, in traditional health care contexts, and in youth recreation centres.

## Background

In order to create effective interventions to prevent mental health problems, we need to know more about who these interventions work for and who is attracted to them [1]. This is especially the case for young women aged 16–24, whose mental health problems have increased significantly in recent decades [2]. If untreated, these problems may linger into adulthood and give rise to a cascade of related difficulties [3]. Relationship quality is important for girls’ and women’s mental health [4, 5]. Relationships can be established through mentoring. Mentoring relationships have the potential to promote developmental assets in young women [6], including improving relationships with others [7, 8], which help these young women thrive as they approach adulthood [6]. In this study, we examine female relationship formation from a protégé perspective in formal mentorships. The study has the potential to provide deeper understanding of an important mechanism that can lead to strengthening young women’s well-being.

Mentoring is an intervention strategy that involves a structured and trusting relationship conducive to establishing a powerful emotional bond between the mentor, the more experienced person, and the protégé, the less experienced [9]. In this study, we adopt a relational-cultural theory approach to the mentoring process [5, 10, 11]. The relational-cultural theory perspective extends our view on mentoring compared to the traditional adult mentoring perspective which describes a one-directional, hierarchical process. Instead, this perspective includes interdependent and mutual processes that result in a full range of relational outcomes for both the mentor and the protégé. The original development of relational-cultural theory was based on women’s behaviours and characteristics, and was used primarily in a therapeutic context. Scholars have now begun to apply the theory to other contexts, including mentoring of college-aged women [12]. The theory posits that women begin to grow, learn, expand, and gain a sense of meaning through relationships with intimacy and emotional connections [11]. Relational-cultural theory holds that mutually empathic healthy growth-fostering relationships include and generate the “Five Good Things”: energy, knowledge, movement, self-worth, and a desire for more connection [5]. Several growth-fostering qualities of relationships have been identified in relational-cultural theory, including mutual empathy (as defined by perceived mutual involvement, commitment, and attunement to the relationship), authenticity (the process of acquiring knowledge of self and the other and feeling free to be genuine in the context of the relationship), empowerment (the experience of feeling personally strengthened, encouraged, and inspired to take action), and the ability to deal with difference or conflict [5].

Sustaining mentoring relationships can be challenging [7, 13]. The psychotherapy literature can be a useful source for understanding how a mentor may act to enhance conditions suitable for developing a growth-promoting mentoring relationship [14]. Psychotherapists characterized as being understanding, accepting, empathic, warm, and supportive, and who do not blame, ignore, or reject their clients, are more likely to have successful results [15]. Also important are dependability, benevolence, responsiveness, and the capacity to convey confidence in their ability to help [16]. Thus, mentors who are able to be empathic and authentic, and convey unconditional positive regard to their protégés, should be more likely to develop an emotional bond and a collaborative structured protégé-focused relationship [14].

Successful mentoring relationships with girls and women are characterized by authenticity, empathy, engagement, empowerment, companionship, collaboration, connectedness, mutuality, and trust [12, 17–24]. Female mentoring relationships which last at least a year have shown promising outcomes [21, 25, 26].

Establishing a trusting relationship, negotiating understanding and meaningful interaction, and preparing for termination have been highlighted as salient processes in the mentoring relationship [27]. At the beginning of the relationship, both the mentor and the protégé may experience some uncertainties and challenges. Unfulfilled expectations, disappointments, pragmatic concerns, and common frustrations often emerge during the early, vulnerable stages (32). Hence, authenticity and empathy are particularly important at the beginning of the relationship, when the mentor must carry the load to achieve continuity [22]. The most successful mentoring relationships are those that evolve from a routine meeting into an enjoyable experience that both the mentor and protégé look forward to and expect to last for a long time [22]. To summarize, there is evidence that the nature and quality of the mentoring relationship are of greater importance than structural components, such as frequency of contact or matching of gender and ethnicity in mentor-protégé pairs, especially when the protégé is a young women [12].

### Gaps in knowledge

Although several studies, many using quantitative data, have examined the outcomes of mentoring [9], we know little about the relational process of mentoring — that is, the nature, quality, and course of mentoring relationships — on the basis of qualitative data [17, 28]. Moreover, we know little about how mentors can be present and helpful in challenging times for young women; this is one indication of a successful mentor-protégé bond [29], and more knowledge of this aspect can help inform the training of mentors [8]. Few studies have examined mentoring programs targeted specifically at female populations, and even fewer have focused on late-adolescent and emerging-adult women, among whom mental health problems increase drastically [2]. Further, not all members of a given gender are the same, and it is of value to examine which young women benefit from interventions and/or are attracted to participate.

This study was based on a Swedish organization working with mentoring aimed at young women. The Girls Zone is one of the biggest non-governmental support organizations working with female mentoring programs for young women in Sweden [30]. It arranges relationships between protégés (young women aged 12–25) and mentors (women ten years older than their protégés). Mentors are engaged in mentorship as volunteers, and are welcome regardless of their formal educational level. The organization is open to all young women who perceive a need for a fellow human being. The stated goals of the program are to prevent mental health problems, promote equality, and prevent drug abuse by strengthening young women’s self-esteem, self-confidence, and trust [30]. Close, regular, and mandatory support of mentors means that any suicidal ideation expressed by the protégés will be reported to the program manager and suitable action will be taken. The approach used is psychosocial mentoring, which prioritizes interpersonal relationship development.

Our study differs from previous studies in several ways. We listened to the voices and histories of the young women, to learn about their descriptions and experiences of the relationship formation process. The mentoring program also has several differences from other mentoring programs that have been previously studied. First, all young women contacting the organization are offered a mentor; it is a universal female mentoring program with no exclusion or inclusion criteria. Second, there are clear rules about the contact between the mentor and protégé. The mentoring program has a time limitation of one year, in contrast to other programs and studies (e.g., [22, 23, 31]). The Girls Zone recommends that the dyads meet every two weeks for about one and a half hours each time, over one year. Except for these meetings, no contact is allowed, which is also in contrast to other programs and studies (e.g., [23]). Third, the mentor and protégé are matched only with regard to age, with a ten-year difference within each dyad; this is in contradiction to the mentoring literature suggesting that a matching process focusing, for example, on the interests of the mentor and protégé is critically important (e.g., [32]). Fourth, there is no parental involvement, meaning that young women may participate without their parents’ knowledge, again in contrast to other programs [33]. Finally, the protégés can restrict their identifiability, to both their mentor and the organization, except with regard to name and e-mail address.

In this study we used a combination of quantitative and qualitative data, collected via self-report questionnaires and in-depth interviews, to examine the formation, development, and closure of female mentoring relationships between protégés and mentors participating in The Girls Zone’s mentoring program from the perspectives of the protégés. We used a relational-cultural theory perspective as a guide to understanding the relational process between the female partners. By doing this, we hoped to develop a more nuanced understanding of the ways these processes promote relation-based mentorship. Two research questions were posed: (1) What characterizes the female protégés attracted to the mentoring program in terms of demographic and psychological characteristics? (2) How does the relationship develop between the protégés and the mentors?

## Methods

### Participants and procedure

**Fig. 1:**
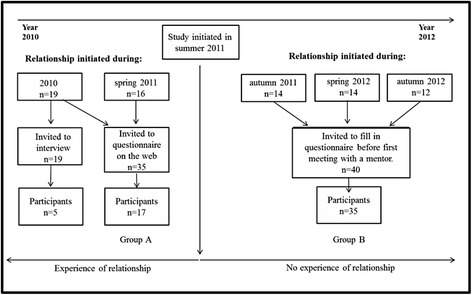
Total sample invited to participate in the study and numbers of participants included

Young women who had contacted The Girls Zone during 2010–2012 in order to find a mentor (*n* = 75) were invited to participate in the study. The participants included both protégés who had an ongoing relationship with a mentor (Group A) and protégés who were about to initiate a relationship with a mentor (Group B) (Fig. 1). This diversity in the duration of relationships gave us the opportunity to obtain a broader and more nuanced picture of the program.

The data collection included both interviews and questionnaires. First, we informed the participants about the study. Participants who were to be interviewed were given information both orally and in writing, while survey participants were given information in writing. After the participants had received the information, we asked them if they were willing to participate in the study. We only included young women who actively agreed in written and verbal consent to participate in the study. In accordance with the Swedish legal rule of research [34], we only obtained informed consent from the young women and not from their parents, given that all of the young women were older than 15. The study was performed in accordance with the Declaration of Helsinki and approved by the Regional Ethical Review Board at Uppsala University (2011/212).

#### Quantitative survey procedures

Sampling was consecutive from August 2011 to December 2012. Both groups of protégés (A and B) were invited to respond to a self-report questionnaire. The program manager of The Girls Zone made initial contact with the protégés. Group A received an e-mail from the program manager with an invitation to fill in a questionnaire on the web, and Group B were asked to fill in a questionnaire when they met the program manager in the organization for the first time. Both groups of protégés (A and B) received information written by the research group (the authors of this study). Likewise, the program manager received a manual with instructions for data collection from the research group. The e-mails to protégés also had cover letters with information about the study and how to contact the research team. A total of 52 participants (69 %) responded to the questionnaire, with ages ranging from 15 to 26 (*M* = 18.1 years, *SD* = 2.8).

#### Qualitative procedure

Young women who initiated a relationship with their mentor during 2010 (all included in Group A) were also invited to take part in an interview (Fig. 1). The program manager sent e-mails to eligible protégés, including information written by the research group. Five protégés out of 19 were interviewed; one further protégé showed interest in an interview, but practical obstacles precluded this. The duration of their relationships ranged between 9 and 15 months. Two of the authors conducted the five semi-structured interviews separately. The interviews lasted 40–90 min each, and focused on the organization, the specific mentoring program, the relationship between the dyads, and the personalities of the young women. All interviews were tape-recorded after approval from the protégés. The interviewees were assigned pseudonyms by which they are referred to in this article.

A mixed method was used to triangulate, complement, develop, initiate, and expand the study [35, 36]. For example, we used information from the interviews to identify the form and content of the items to be used in the quantitative study (i.e., the survey questions). A sequential mixed-method approach, QUAL-Quan, was adopted to provide a holistic understanding of the specific situations of the protégés, to ensure high external validity, and to allow us to study the variation and context [35, 37]. All protégés who participated in the study received a movie ticket as a reward.

### Measures

Most measures in the questionnaire have been used previously (e.g., [38–42]).

#### Sociodemographics

Sociodemographic factors were measured using five items: country of birth, sibling or not, living with parents or not, employment status, and perceived economic situation (“How do you rate your economic status in relation to others of your age?”).

#### Health status

Subjective health was measured using the item “How do you rate your general health?” on a response scale ranging from 1 (“*Very good*”) to 5 (“*Very poor*”). Anxiety levels and mental suffering over the past six months were measured using three items: “Do you deliberately harm yourself with a sharp edge?”, “Do you receive pharmacological treatment for mental health problems?”, and “Do you have feelings that you do not want to go on living?” The response scale ranged from 1 (“*Every day*”) to 5 (“*Never*”). Feelings of loneliness over the past week were using the question “Have you felt alone?” with a response scale ranging from 1 (“*Never*”) to 5 (“*Always*”).

#### Interaction with others

The protégés were asked to report on whom they talked with if they were feeling anxious or worried, using one open-ended stem question with fifteen response options covering *parents, siblings, school nurses,* and *nobody at all*. For the analyses, the scale was condensed to include eleven categories.

We also asked the participants how they had obtained information about The Girls Zone. Eight response options were provided, including *parents, school nurses,* and *the web*. A similar question was posed concerning how the young women made contact with the organization, with five response options including *by myself*, *with the help of my parents*, and *through the school nurse*. Both of these questions also permitted open-ended responses.

### Data analysis

Both qualitative and quantitative data were used, and we analysed the information both statistically and through content analysis.

#### Survey data

First, we compared Group A with Group B in terms of variables concerned with sociodemographic factors and health, using independent samples t-tests. No differences were found regarding health, well-being, or age, except the expected differences related to age such as living with parents or not, and employment status (Fs ˂ 2.71, ps ˃ .10). Second, we compared women who were 17 or younger with those 18 or older on variables concerned with sociodemographic factors and health, again using independent samples t-tests. Despite the large range of ages (15–26), again only the expected differences depending on age were found, such as living with parents or not, and employment status (Fs ˂ 2.31, ps ˃.10). Accordingly, we did not consider the group division further.

#### Interviews

**Fig. 2:**
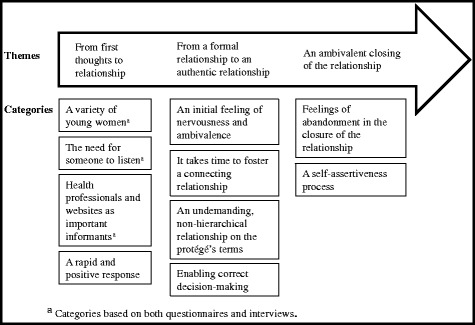
Findings from the integrated analyses

We analysed the data from the interviews using the technique of inductive qualitative content analysis [43]. The two authors who performed the interviews transcribed the interviews verbatim, and then read through the transcribed interviews several times to obtain a sense of the whole. Triangulating analysis was used, meaning that initially these two authors analysed the same interview. First, meaning units were identified, each consisting of a constellation of words relating to the same central meaning [43]. The meaning units were then condensed, with a description close to the text, and each condensed meaning unit was labelled with a code on the basis of its content. All the codes were clustered into categories with the same content, and the categories were labelled, with the descriptive labels again kept close to the content. Contrastive comparisons between the codes and categories were made, and a schema was drawn up. Following this, the rest of the interviews were analysed by one of the authors. New codes that emerged were discussed by all authors and consensus was reached regarding categories, with all codes included. Themes within the data became apparent, as there were distinct groups of categories which had similar meanings or were about similar topics. Three themes were identified from a time perspective to cover the formation, development, and closure of relationships (Fig. 2). Several steps were included in the analysis to ensure trustworthiness [44], including triangulating analysis, a debriefing session with the organization’s program manager (in which the findings were presented and found to be in line with the manager’s own experience), and peer-examination (i.e. a discussion of the process and findings with impartial colleagues).

## Results

The results are described in terms of two questions — “What characterizes the female protégés attracted to the mentoring program regarding demographic and psychological characteristics?” and “How does a relationship form between the protégé and the mentor?” — and presented on the basis of a time process from before the start of the relationship until the end of the relationship. Results from both the questionnaires and the interviews are presented. Figure 2 shows the themes and categories, and which categories are based on both interviews and questionnaires.

### From first thoughts to relationship

Descriptive characteristics of the participants are presented in Table 1. The process from first contact until initiation of the relationship with the mentor is described below on the basis of both the interviews and the questionnaire data (Fig. 2).

**Table 1:** Health status and communication patterns

Measure and variable	*n* (%)
Self-rated health (*n* =48)	
Very good or good	18 (38)
Neither good nor bad	18 (38)
Poor or very poor	12 (21)
Non-suicidal self-harm behaviour (*n* =50)	
Once a week or more often	5 (10)
Seldom or never	45 (90)
Pharmacological treatment for mental health problems (*n* =50)	
Once a week or more often	12 (24)
Seldom or never	38 (76)
Feelings of not wanting to go on living *(n* =49)	
Once a week or more often	17 (35)
Seldom or never	32 (65)
Feelings of loneliness (*n* =50)	
Often or always	29 (58)
Never, seldom, or sometimes	21 (42)

#### A variety of young women

Results from both the questionnaires and the interviews are presented to address the question of what characterizes the female protégés attracted to the mentoring program, in terms of demographic and psychological characteristics.

The participants were between 15 and 26 years old. The majority (87 %) were born in Sweden, and 89 % had siblings. The majority (87 %) attended school, while the rest were employed (7 %), unemployed (4 %), or on sick leave (2 %). Almost 40 % of them lived with both their parents. Of the remainder, 42 % lived with one parent, or with two parents in alternation: some lived only with their mothers, some lived only with their fathers, and others alternated between living with their mothers and living with their fathers. Finally, 10 % of protégés lived alone, and 10 % lived together with someone other than a parent.

Health status varied among the protégés. Although most regarded their health as very good or good, or neither good nor bad, 21 % perceived their health to be poor or very poor (Table 1). Some of them reported non-suicidal self-harm behaviour: 10 % deliberately harmed themselves with a sharp edge once a week or more often, 24 % had received pharmacological treatment for a mental health problem, and 35 % had had the feeling that they did not want to go on living during the last week. More than half of the protégés (58 %) had feelings of loneliness over the past week. When the protégés needed to talk to someone, they usually talked with a female friend (65 %) or their mother (63 %). Other persons they could talk to were professionals (31 %), male friends (31 %), fathers (25 %), teachers in school (14 %), other relatives (21 %), partners (16 %), siblings (14 %), and others (10 %). One in ten reported that they did not have anyone to talk to.

The protégés in the interviews were aged between 18 and 24; attended school, were employed, or were on sick leave; and described themselves in words and phrases such as “cheerful”, “considerate”, “kind”, “active”, “analytic”, “positive”, “honest”, “find it easy to laugh”, and “social”. When not in school or work, they spent their time on homework, part-time work, and leisure pursuits such as physical training, personal development, and music.

#### The need for someone to listen

Despite different kinds of problems and degrees of significance, all protégés expressed a need to talk with someone who would listen, preferably an adult, to help them handle circumstances in life and to make them feel better. One said:

[…] in order to find a way back to myself. I was tired of … I didn’t recognize myself. So, I was just tired of being sad. Just wanted to get into another world in some way. (Kate)

The protégés in this study were navigating different complicated and compromising life circumstances that were their reasons for contacting the organization, such as eating disorders, depression, stress and pressure, social anxiety, and loneliness. They had also wanted to talk about things that they did not want their family or friends to know about, or just about family, friends and school in general. The results from the questionnaires and the interviews were in agreement.

Some protégés had been involved in the traditional health care system before they made contact. However, they still felt the need for a person to talk to, since the support they received from traditional health care was unsatisfactory. In some cases, the need to meet someone had also been expressed by others, not just by the protégés themselves; for example, by a school nurse who suggested that the young women should contact The Girls Zone.

#### Health professionals and websites as important informants

We first present results from the questionnaires, and then from the interviews. Four out of ten protégés had obtained information about the organization from professionals such as school nurses or school welfare officers (Table 2). The Internet seemed to be an important information source too, with one in four protégés having obtained information on the Internet, friends, parents, and advertisements were also good informants about the program. Most of the young women who became protégés made contact with the organization on their own after finding out the initial information about the program.

**Table 2:** The route to the Girls Zone

Measure and variable	*n* (%)
From whom did you hear about the organization?^a^ *(n = 52)*	
From professionals	21 (41)
From the Internet	13 (25)
From friends	11 (21)
From parents	7 (14)
From advertisements	7 (14)
How did you get in contact with the organization?^a^ *(n = 52)*	
By myself	39 (75)
With the help of professionals or teachers in school	14 (28)
With the help of parents or friends	6 (12)

^a^The participants’ responses could be coded under more than one subtheme

Again, the results from the interviews corresponded to those from the questionnaires. The first contact with the organization was made on the basis of information from professionals, the Internet, or friends. These agents recommended the program and provided information that the protégés were interested in researching before using the organization’s website to make first contact. On this website, the protégés obtained information about different programs, and read true stories about mentors and protégés concerning their relationships and the meanings of them.

#### A rapid and positive response

When the protégés contacted the organization for the first time, they appreciated the quick response they received. For those with experience of traditional health care, this was unexpected, and they had the surprising feeling that someone really wanted to listen to them and took their problems seriously. Hence, they gained trust in the organization from the very beginning. Regardless of whether they sent an e-mail or made a phone call, they very quickly obtained an introduction to a mentor.

I mailed The Girls Zone. Got a reply after about 10 min. Then, it took just a week at most before I met my mentor for the first time. So it went really quickly. (Adele)

### From a formal relationship to an authentic relationship

How does the relationship between the protégé and the mentor form? The findings here are based on interviews related to the development and the formation of relationship.

#### An initial feeling of nervousness and ambivalence

Before a pair of strangers came together for the purpose of developing a one-year relationship, expectations varied among the protégés. The narratives included both neutral beliefs and beliefs that the relationship would not help. For some, this was dependent on previous experience of professional health care.

[…] I just felt that whoever I talk with won’t play any role. It won’t work. (Evelyn)

At the same time, there was a sense of gratitude for the opportunity to seek this support. The narratives also expressed anxiety and wondering about what would happen and how the mentor would perceive her young protégé. There were expressions of embarrassment, and a lack of insight into how to talk about one’s mood.

I was grateful, very nervous, and tense about what would happen or what she would think of me, how she would perceive me. Because I felt pretty good here, but God, but I felt a bit stupid, and that I might react badly to something when I didn’t know what it was. I felt a bit ridiculous in a way, but what would she think of it. There was a lot of nervousness, I think. (Kate)

At the beginning of the relationships, there were barriers to meeting because the protégés felt themselves to be in an abnormal situation. However, there was still a willingness to meet.

The first times I just felt “I don’t want this, no, I don’t want to do this”. I was like this, backed off, felt like it was really unpleasant, something unusual, but it was also something I needed as well…so I forced myself to the next meeting. (Kate)

Having similar personalities in the dyads was not regarded as necessary to create a trustful relationship. Empathetic accommodation on the part of the mentor was of much greater significance.

We really clicked the first time we met. We are quite different, and appear quite different as people, but […] I can say anything to her, she understands. If I say “I can’t manage to meet”, she understands why. (Adele)

#### It takes time to foster a connecting relationship

At the beginning, the protégés sometimes had a fear of relaxing, opening up, and talking. Then, the dyads started to share personal experiences and expectations. As the pair grew to know one another better, they felt trust and closeness, and the protégé talked about anything she wanted. As a result, many feelings flared up, which needed to be processed.

[…] at the beginning, a lot of anxiety was created, because I started digging up everything that had been repressed for several years. So, at the beginning, there were a lot of tears, severe anxiety after the meetings, or when I got home and started to think about it all. (Kate)

Despite these feelings, the protégés waited expectantly for their meetings; as one of them expressed it, “It will be fun to meet her!” After some meetings, support went beyond the protégés’ expectations.

#### An undemanding, non-hierarchical relationship on the protégé’s terms

When the dyads met, the protégés got a break from the world outside; this was their time, focusing only on them, with an opportunity to talk about feelings and any problems to be solved. As a result, the protégés could be in a better mood at the end of the meeting. One of the reasons they found it helpful to talk to a mentor was that the mentors and protégés did not previously know one another. The protégés decided for themselves what kinds of information about their lives, and how much, they wanted to reveal to their mentor.

I choose exactly what the mentor gets to know about me, and I don’t need to tell her anything, but I can also tell her everything. (Kate)

This arrangement set the stage for a relationship in which both parties felt they were free to speak, and share their thoughts and feelings. The protégés had the feeling of not being judged according to whom they were as people, but only on the basis of the things they chose to share; they greatly appreciated this approach, in which the mentors only received information about the protégés from the protégés themselves.

#### Enabling correct decision-making

In the face-to-face meetings, the protégés met mentors who cared about and believed in them, who saw them, and who listened to them. The protégés were treated with respect, and appreciated having someone to talk to, not only as a friend but as a person who gave them perspectives and shared their points of view. Specifically, the protégés appreciated that their mentor gave suggestions about how to handle various situations. All five protégés interviewed stated that their mentor was a good listener and came up with very good advice. The mentors’ attitude to the protégés was described as positive, strengthening, and promoting. The protégés also mentioned that the mentors did not have the role of deciding what they should do, which was much appreciated.

She isn’t someone who says: “This is what you must do” or “That’s how it should be”. She said: “What do you feel yourself?” She came up with ideas, but still about things that I thought were relevant, not things that an expert or a professional would suggest. She came up with ideas that gave me insight into: “What are you really up to? […]”. (Evelyn)

The mentors encouraged their protégés to open up and talk about everything, no matter what. As a result, the protégés shared things they would not dare to report to others such as youth guidance officers or friends.

### An ambivalent closing of the relationship

Close ties were developed between the dyads, so that the mentor was said to know the protégé better than her friends did.

She knows more about me than most of my friends do […] She probably knows me better. (Kate)

The relationship grew into something more significant than just a conversation between two young women. In part, this showed that the mentor could break some of the rules made by the organization, for example by visiting the protégé in hospital.

#### Feelings of abandonment at the closure of the relationship

The protégés’ narratives revealed a mutual wish between protégés and mentors to stand by each other’s side in the future. According to the protégés, mentors wanted to take part in the positive change even after closure of the relationship in the organization’s directory. However, continuity of the relationship was something that the dyads themselves had to decide upon. The duration of one year was presented to both partners at the initiation of the relationship. Despite this, the narratives showed feelings of despondency, sadness, and anxiety when the relationships were ended.

Well, are you just going to leave me now? You can’t do that. What? I can’t be without her. No, I don’t even want to think about it. It feels like half of me is just disappearing […] it’s going to be really strange. I’ll be really sad. What? Last time we meet. No, damn it! (Adele)

Replacing a mentor with a new one would feel strange and could lead to a disappointment, with the feeling that she was not as good as the previous one. Instead, the protégés thought ahead and used what they had learned during the relationship in order to manage their life situations.

#### A self-assertiveness process

During their relationship, the protégés were strengthened as individuals. However, the journey had sometimes been a difficult one.

The time I’ve been meeting her has been both the best and worst time in my life. […] it has torn down so many walls, and I have come to terms with so much about myself that has been pretty unpleasant, but it has been wonderful that it went so well at the end. But that time before I crossed the threshold was terrible. I didn’t recognize myself. So much was happening inside me. It was really nasty. (Kate)

The meetings with the mentors allowed the protégés to practice expressing themselves and putting their feelings and thoughts into words. The result was that they dared to open up and talk to other important persons as well as their mentors. With the help of their mentors, they had processed a range of different complicated life circumstances, which meant that their experience of the problems at the end of the relationship was less than it had been at the beginning. They accepted themselves, respected themselves, and got to know themselves better, and they became satisfied, motivated, and energetic. One protégé expressed this as follows.

I feel much happier anyway now, so it feels much easier. Doesn’t feel as tough. (Sienna)

They also obtained tools to help them feel ready to try to manage themselves, and to keep on working to continue feeling quite well.

I thought it was crazy that it was just one year. That it was too short. But then I think that, just for me, it was pretty good that it wasn’t any longer. That I was forced to take a step forward. That I shouldn’t just hang around in the same place. Instead, I feel I’m moving on. (Jenna)

With a positive experience of the mentoring program, the protégés could consider themselves as potential mentors of other young women in the future.

That I would really be able to think of doing, obviously. Now that I’ve been a protégé, and know how much it means to me. (Adele)

## Discussion

In this study, we examined the formation, development, and closure of face-to face same-sex relationships in a one-year mentoring program for young women aged 15–26. The main findings indicate that, as a group, the protégés were heterogeneous and did not necessarily differ from the majority of young women of the same age group. Many of the protégés acted individually, or were recommended by professionals to meet and talk to an older women. These meetings with listening, undemanding, and non-hierarchical mentors developed into engaged, empathic, and authentic relationships which were in a good position to continue even without the organization’s involvement after one year’s implementation.

We posed two specific questions in the study. The first was: “What characterizes the female protégés attracted to participate in the mentoring program in terms of demographic and psychological characteristics?” To answer this question, we used data from questionnaires and interviews, and examined the characteristics of the young women taking part in the program (i.e., the protégés).

The results indicated a large variation among the young women, who seemed to lack any clear defining characteristics that made them stand out in comparison with the normal group of women of this age in Sweden. The reasons why protégés considered contacting the Girls Zone seemed to be quite similar to the “disconnections” which according to relational-cultural theory [5, 10] result from a lack of mutual relationships: diminished sense of well-being, loneliness, confusion, eating disorders, and non-suicidal self-harm behaviour. Despite the fact that most of the protégés reported that they had someone to talk to, they still expressed feelings of loneliness, as well as a need to meet and talk with a person who can listen, and definitely wants to listen. Accordingly, there seemed to be an unmet need for mutual engaged, authentic, and empathic relationships among the protégés in their daily lives, expressed as important in relational-cultural theory. It is possible that there are young women who have many social contacts but few face-to-face relationships, or parents who are busy and do not have enough quality time for their daughters, since the results show that many of those in the present study were living away from both parents.

The second question posed in the study was: “How does a relationship form between the protégé and the mentor?” We used the questionnaire and interview data to address this question. One unexpected finding was that some of the young women were referred to The Girls Zone through professionals such as school nurses. For young women still attending school, professionals in school health care are the most accessible place to seek help with perceived problems, beside parents and peers. It would be of interest to discover why these professionals do not believe that they are the right person to support these young women, and to investigate why the young women themselves regard the support they receive from traditional health care as unsatisfactory. Access to counsellors is insufficient in Sweden [45], and young women’s developmental challenges seem to fall through the cracks of different support services because their needs do not entirely match the inclusion criteria [46]. Furthermore, the traditional model of therapy suggests a role for a non-expressive detached therapist trained to distance themselves from any strong feelings of their own. This is not in accordance with the findings of our study, previous research, and the advocates of relational-cultural theory; rather, young women want an authentic relationship with deep respect and mutuality [5]. Previous research has shown that young people are clear about wanting to know their school nurse and to create continuity [47]. Moreover, the protégés in the present study used the Internet as an important channel for information about the program; in other words, they showed pro-active self-care in seeking out the program, despite ambivalence over whether a mentor would help them feel better. This is in accordance with relational-cultural theory, which posits that there is a powerful force behind the movement toward connection, and a desire to contribute to others. Moreover, the mentors themselves made contact because they wanted to become involved in relationships with young women on a voluntary basis. This meant that both mentors and protégés had a strong desire to make the relationship work. Some of the factors that Spencer [48] views as critical in the failure of mentoring relationships, such as low degree of motivation, can be seen as having been eliminated in the relationships described in the present study.

The protégés’ first contact with the organization was a positive experience for them, with a rapid and positive response creating an immediate feeling of trust in the organization. After some meetings characterized by ambivalence and nervousness, an authentic relationship was developed, characterized by a mentor who listened to the protégé and treated her with respect. Mentor and protégé having the same personality was not crucial to feeling an intimate connection. Rather, understanding and empathy from the mentor were of significant value, which is in accordance with a previous study [12] but contradicts previous research showing the importance of the match between the mentor and the protégé for ensuring that the protégé’s needs are met [32].

Our results are in line with key elements of relational-cultural theory [5] and of psychotherapy [15, 16], which Spencer [14, 48] also suggests may be transferable to mentoring relationships. Mentors need to show understanding and acceptance of their protégés, and must also show empathy and warmth, and be supportive. Our results also show that, developing a relationship with a previously-unknown and non-judgmental person was perceived by the protégés as a positive way to help them open up and feel the authenticity of the relationship. The current findings suggest that not needing to achieve or perform and the absence of certain demands, such as sharing information about themselves, were valued features of the relationship, and were perceived as offering a break from what is the norm in other aspects of life. The mentor was not seen as an expert, even if the protégés sometimes asked for advice. These requests for advice were met with counter questions such as “What do you feel yourself?” This way of replying on the part of the mentor was appreciated by the protégés, and strengthened them in getting to know themselves better. The young women’s desire to obtain advice about their problems also shows a wish for mentoring to not only include psychosocial mentoring, in which interpersonal-relationship development is a priority, but also to have an element of instrumental support, like role modelling and coaching; this is consistent with the previous literature [20, 21, 23].

Based on previous research showing that female protégés prefer and are more satisfied in mentoring relationships which last longer than a year [26, 49], and also in accordance with the relational-cultural theory that advocates women’s lifelong relationships [5], the restriction of the relationships in this program to one year can be regarded critically. However, the protégés were able to decide for themselves whether or not to continue to see their mentors, separate from the organization’s involvement, provided that the desire was mutual (from both protégé and mentor). The narratives indicate that both protégés and mentors expressed a desire to continue the relationship beyond the initial year. The protégés did not want to be left alone; they wanted to continue to meet their mentors so they could continue to experience the positive change that had begun in their lives. This desire to continue the relationship is an outcome that mirrors the fifth Good Thing from relational-cultural theory: a desire for more connection [5]. Moreover, our findings concerning the relationship process showed personal development of the protégés according to all the Five Good Things in relational-cultural theory, including more energy, action, knowledge, sense of worth, and sense of connection in the relationship.

The most rigorous research suggests that mentoring approaches yield only modest effects for young people [9]. The narratives in this study spoke of the meaning of the relationships at the moment the protégés met their mentor; that is, the meeting made them feel better afterwards, when they had put all their feelings and thoughts into words and reflected upon them with another person. The formation and development of the mentoring relationships had resulted in relationships between two women characterized by authenticity, empathy, connection, and closeness, according to previous research [12, 23, 28]. Furthermore, according to Lerner and colleagues [6], our results conform to the “Five Cs” of positive youth development: competence, confidence, connection, character, and caring.

The theoretical framework for the mentoring program is not made explicit by the organization itself, but we considered relational-cultural theory to be a good frame for understanding these female relationships. One issue in the theory is how to create the societal context within which growth-producing relationships can flourish in the manner of mutual empowerment. Reagan-Porras [29] has previously posed the question of whether mentoring programs target young people in the ways that they state are significant to them. The results of the present study indicate that adolescent and emerging-adult women can establish an authentic relationship with a non-kin adult woman ten years older if the relationship is undemanding and non-judgmental on the protégé’s terms. This has a role to play in giving young women the tools to deal with challenging and intractable experiences in the future. The protégés in our study used the relationship to navigate the bumpy road of adolescence and emerging adulthood. The program also counters silence among women, and can lead on to contacts beyond the mentor-protégé relationship [5, 8, 9]. Hence, for those who need further help, a mentoring relationship can be seen as an aid to seeking professional help and as a supplement to formal mental health treatment [50].

The aim of the mentoring program, as stated by the organization, includes the prevention of mental health problems. It is thus worth asking why we saw no differences in mental health between Groups A and B, even though several relationships in Group A had been completed when data collection was carried out. No baseline assessments were made, so we do not know the mental health status among those in Group A before they were introduced to a mentor. Their mental health may have improved. Another explanation may be that, although the primary goal of the intervention is to prevent mental health problems, it is not a treatment. The intention is to create the conditions to build a one-year relationship. During this period the organization offers support that facilitates the relationship, including matching, training, mentor support, and technical support. The organization is small-scale, and is intended to remain so in order to maintain high quality. According to the program manager, several relationships continue even when a relationship is terminated within the organization’s responsibility. Based on the results of this study and previous research on women and mentoring, showing that women appreciate relationships of longer than a year, and that it takes time for women to establish relationships with close ties [26, 49], it is likely that relationships like these promote developmental assets useful in the protégés’ future lives. We did not examine the long-term effects of relationships in this study, but will do so in the future. Furthermore, previous research reveals that many problems in young women decrease by themselves with advancing age (e.g., [51]). We do not know how these young women would have reported on their self-rated health without this intervention. It is possible that their problems would have been more serious than our quantitative data show.

Our findings indicate that the mentoring program is better able to meet the needs of protégés than the support and help that traditional health care has to offer. Moreover, health professionals are important informants about the program. Comparing the mentoring program with a traditional adult perspective and relational-cultural theory perspective on mentoring, there are several similarities between relational-cultural theory and the mentoring program. These include the facts that both mentor and protégé contribute to the development of the relationship, and that it is a non-hierarchical relationship where both the mentor and protégé have increased ability to function in an interdependent context.

### Limitations and strengths

This study has some limitations. First, only 52 questionnaires and five interviews were included. On the one hand, this may be seen as too few; but on the other, the results from the interviews were validated against, and found to correspond with, the data from the questionnaires. Second, the sample represents protégés from the region of Stockholm, the capital of Sweden. It is possible that the results might only reflect conditions in a large city. Third, the participating protégés represent a group of young women who were satisfied with the program. However, there were few protégés who had started a relationship but did not follow the plan for the one year stipulated. Forth, we did not use a procedure which allowed us to follow-up with participants who reported suicidal ideation. Even though participants had mentors and were enrolled in an intervention, we cannot be sure that the mentors or the program staff were aware of the concerns. However, our research and question about suicidal ideation is not likely to harm the participants as previous research has demonstrated that asking about suicidal behavior does not exacerbate suicide prone individuals’ suicidal thoughts and behavior [52]. In fact, it has been suggested that it might be helpful to these individuals [52].

Despite these limitations, the current study has several important strengths. First, this is the first study we know to have examined the relational process, especially among young women aged 15–26. Second, we used a combination of quantitative and qualitative data, collected via self-report questionnaires and in-depth interviews, to achieve a deep and holistic understanding of the specific situation of the protégés. As a result, we handled the limitations of the two methods at the same time, since the quantitative and qualitative methods overlap in their intent [35, 36].

Third, one group of protégés participating in the study had experience of the mentoring relationship, whereas the other group had just started. Irrespective of the time status of their relationship, both groups of protégés expressed optimism about the program. The difference in duration of relationships between participants gave us an opportunity to obtain a more nuanced and broader picture of the mentoring process.

Fourth, Article 12 of the UN Convention on the Rights of the Child asserts children’s right to express their points of view [53]. The interviews allowed the young women to express themselves, and let them talk about their everyday lives and their experiences of the relationship.

### Practical implications

A practical implication of these findings may be that female relationship mentoring training and tutorials need to focus on teaching the mentor to listen actively, show respect, and pay attention to what gives the protégés strength, hope, and desire, with a feeling of empathy, engagement, and authenticity in a non-hierarchical relationship. This can be adapted for use in several other arenas where young women create authentic relationships with older individuals, including schools, traditional health care, and youth recreation centres. Further, organizations need to create the preconditions for female face-to-face relationships to be realized. If this happens, and if the relationship is offered the conditions to grow over a year with the help of preparatory education of the mentor, clear frames and rules for the relationship, and regular supervision of the mentor, good conditions are created to develop a relationship that can continue even without the organization’s involvement after one year’s implementation.

### Issues for further studies

While the findings of this study identify the female mentoring program as an intervention worth exploring and continuing, many questions remain unanswered. The forthcoming longitudinal data following the protégés from start to the closure of the relationship and two years after will give a more nuanced and realistic view about the impact of the relationship on mental health among the protégés. Further, based on the current study, we now have information about who the protégés actually are, their needs, and the formation, development and closure of a relationship process in the program from a protégé’s perspective. An analysis of this from the perspective of the mentors using relational-cultural theory is planned. Other aspects of interest include what motivates mentors to be volunteers in this organization, and what the relationship means to them. Finally, the factors for success in this program need to be further explored. These issues are currently being further analysed in an ongoing longitudinal study. Moreover, the quality of the mentoring process is an important concern and future research will consider different challenges including incorporating provisions to ensure that participants reporting suicidality have access to appropriate support.

## Conclusions

Mentorships that are in line with the perspectives of relational-cultural theory meet the relationship needs expressed by the female protégés. Mentor training should focus on promoting skills such as active listening and respect for the protégé based on an engaged, empathic, and authentic approach in a non-hierarchical relationship. These insights have the potential to inform interventions in several arenas where young women create authentic relationships with older persons, such as in schools, in traditional health care contexts, and in youth recreation centres.
